# Lesions involving the insula are associated with reduced appetite and weight loss

**DOI:** 10.1093/braincomms/fcag044

**Published:** 2026-02-25

**Authors:** Wanzhi Lyu, Joel Bruss, Emily R Dappen, Joseph C Griffis, Benjamin Pace, Brandon Neisewander, Kenneth Manzel, Daniel Tranel, Aaron D Boes, Nicholas T Trapp

**Affiliations:** Department of Psychiatry, University of Iowa, Iowa City, IA 52242, USA; Institute for Human Neuroscience, Boys Town National Research Hospital, Omaha, NE 68010, USA; Department of Pediatrics, University of Iowa, Iowa City, IA 52242, USA; Department of Neurosurgery, University of Iowa, Iowa City, IA 52242, USA; Department of Pediatrics, University of Iowa, Iowa City, IA 52242, USA; Department of Psychiatry, University of Iowa, Iowa City, IA 52242, USA; Department of Psychiatry, University of Iowa, Iowa City, IA 52242, USA; Department of Psychological and Brain Sciences, University of Iowa, Iowa City, IA 52242, USA; Department of Psychological and Brain Sciences, University of Iowa, Iowa City, IA 52242, USA; Department of Neurology, University of Iowa, Iowa City, IA 52242, USA; Department of Psychiatry, University of Iowa, Iowa City, IA 52242, USA; Department of Pediatrics, University of Iowa, Iowa City, IA 52242, USA; Department of Neurology, University of Iowa, Iowa City, IA 52242, USA; Iowa Neuroscience Institute, University of Iowa, Iowa City, IA 52242, USA; Department of Psychiatry, University of Iowa, Iowa City, IA 52242, USA; Iowa Neuroscience Institute, University of Iowa, Iowa City, IA 52242, USA

**Keywords:** appetite, body weight, brain lesions, brain−behaviour relationship, structural MRI

## Abstract

Eating-related disorders such as anorexia nervosa, bulimia nervosa and obesity are associated with high rates of morbidity and mortality in the United States. To investigate the neuroanatomical structures involved in appetite and weight change, we employed lesion symptom mapping. 358 patients with focal brain lesions and appetite ratings were recruited, as well as 48 patients with pre- and post-lesion weight records. Partial least squares regression identified a significant association between patterns of brain damage and appetite change (model *R^2^* = 0.13, *P* = 0.006), and the relationship between lesion location and weight change was explored using the proportional subtraction method. The right posterior insula was the peak region associated with both decreased appetite and weight loss, providing new insight into the neural correlates of higher-order appetite regulation and weight management.

## Teaser

Lyu *et al*. demonstrated that damage to specific brain regions relates to changes in appetite and weight after neurological injury. In two distinct analyses, lesions to the right posterior insula were associated with reduced appetite and weight loss, identifying this region as a key contributor to appetite and weight regulation.

## Graphical Abstract

**Graphical Abstract:**
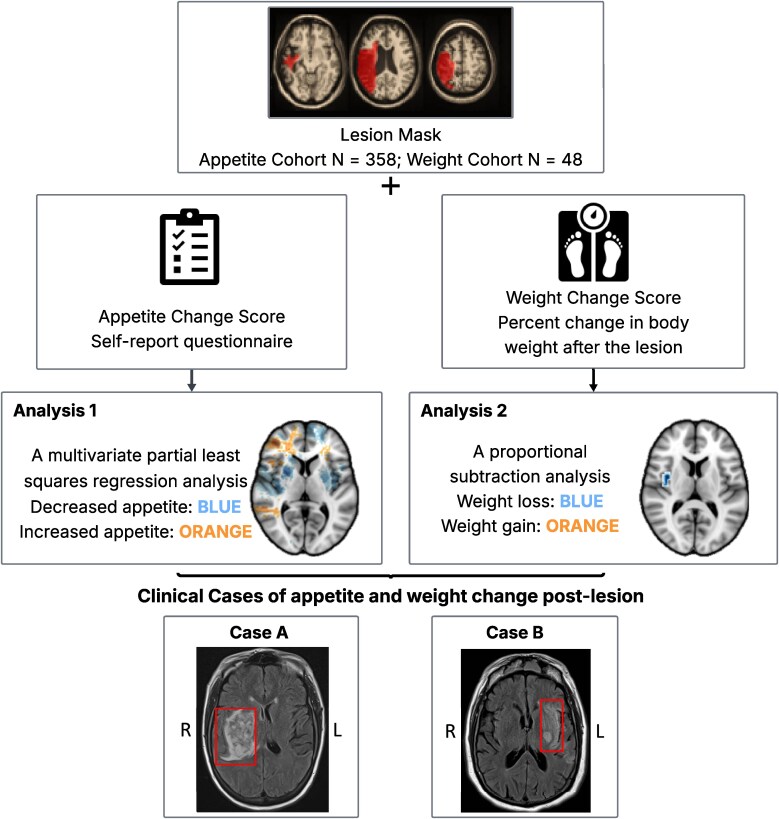

## Introduction

In recent years, the USA has seen a rise in nutrition-related health issues ranging from anorexia nervosa to obesity.^1^ These conditions are often chronic and associated with significant morbidity and mortality.^2,3^ Amongst all psychiatric disorders, anorexia is the condition associated with the highest mortality rate due to medical complications of severe caloric restriction and associated behaviours.^4,5^ Appetite and weight change are also important symptoms and signs in many other psychiatric conditions including bulimia nervosa, major depressive disorder, binge eating disorder and anxiety disorders.^6^ Although many pharmacologic and surgical treatments have aimed to adjust appetite for therapeutic purposes,^7^ the neural circuitry underlying the physiology of appetite regulation, particularly within the forebrain, has been elusive. Large lesion databases provide an opportunity to examine the neuroanatomical correlates of appetite and weight change by exploring the relationship between brain lesion location and changes in these measures.

The temporal relationship between a brain lesion and a subsequent behavioural change provides a significant window to study brain−behaviour relationships, implying the functional necessity of the damaged brain region or its connections for the behaviour of interest. Human lesion studies throughout history have yielded valuable insights into the functional organization of cognition and behaviour within the human brain, contributing significantly to current models of brain function across various neurobehavioural domains such as language,^8^ memory,^9^ attention,^10^ mood^11–13^ and psychosis,^14–16^ amongst others.^12^ This investigative technique, known as lesion-symptom mapping, has evolved and grown in use with access to larger sample sizes and more complex statistical methods. However, to our knowledge, no large-scale lesion studies have examined the relationship between lesion location and appetite or weight change following focal brain injury. The current study includes the largest sample to date to address this question.

Prior neuroimaging research on the neuroanatomical correlates of normal and disordered appetite and feeding behaviour implicates various brain regions, including the insula, the striatum, the hypothalamus and the prefrontal cortex. Insula and striatum dysfunction have been associated with disturbed eating habits in bulimia nervosa,^17^ anorexia nervosa and lesion-associated anorexia.^18,19^ In healthy subjects, ventromedial prefrontal cortex (vmPFC) grey matter volume predicted better dietary self-control.^20^ This variability of findings suggests that multiple brain regions are likely involved in the complex processes mediating feeding behaviour and appetite. Interpreting and synthesizing these findings is complicated by the variability in the study methods, including disparities in the behavioural assessment tools employed, study population demographics and methods for neuroanatomical inference. The challenge of localizing complex behaviours to precise anatomical regions is further compounded by small sample sizes and limited statistical power in work to date. Prior lesion−symptom mapping studies of complex cognitive and behavioural outcomes, such as depression, suggest that large sample sizes are needed to identify associations.^12^ Here, we leverage a large research database of patients with brain lesions, high-resolution neuroimaging data, appetite data and weight change data to overcome some of these challenges.

The aim of this research was to explore the relationship between brain lesions and changes in appetite in a large cohort of patients (Analysis 1), and then to compare the identified regions of interest to those associated with weight loss in a smaller sample of patients with prepost weight data (Analysis 2). Previous literature suggests that the insula and prefrontal cortex are implicated in appetite and weight regulation, as activity in these regions is associated with taste processing, impulse control and craving for rewarding substances.^21^ However, details regarding laterality of findings, regional specificity within the insula and prefrontal cortex and potential involvement of other brain regions are unclear due to a lack of prior lesion studies. To examine these relationships without restricting analyses to predefined regions, we applied an exploratory, whole-brain, data-driven approach to identify brain−behaviour relationships as opposed to restricting analyses to those regions of interest.

## Materials and methods

### Experimental design

Patients were recruited from the Patient Registry of the Department of Behavioural Neurology and Cognitive Neuroscience at the University of Iowa. The study was approved by the University of Iowa Institutional Review Board, and all patients signed informed consent. Each participant was enrolled after the onset of their lesion. For Analysis 1, patients were only included if they had a valid Beck Depression Inventory-II (BDI-II) scale appetite item completed in the chronic phase post-lesion (defined as 90 days or more after the lesion) and a structural MRI scan. The BDI-II appetite item assesses changes in appetite over the past 2 weeks, with 7 response options ranging from decreased to increased appetite, allowing researchers to capture both directions of change. Exclusion criteria were a history of severe alcohol or drug abuse, diagnosed serious mental illness prior to brain injury, medically refractory epilepsy or epilepsy surgery as the reason for the lesion or other diagnosed neurologic disorders unrelated to the lesion.

For Analysis 2, patients were included if they had at least two valid weight records: one recorded within 90 days of the lesion (W1) and one recorded between 90 days and 1000 days after the onset of the lesion (W2), consistent with prior literature on the expected time frame for weight change after a traumatic brain injury.^22^ A minimum time window of at least 30 days between W1 and W2 was required to ensure an adequate amount of time had passed to realize weight change attributable to a behaviour change,^23^ while the 1000-day post-lesion upper limit allowed removal of cases where the weight change time horizon was considered too remote from the lesion onset and thus increasingly less likely to have weight change attributable to the lesion itself.

### Lesion segmentation

Each participant included in the analysis had a focal brain lesion with visible boundaries evident from structural neuroimaging from T1 and T2 sequences on 3T MRI. Imaging for all patients was performed in the chronic epoch (>3 months since onset). The anatomical segmentation of lesion borders was traced manually for each subject and brought to a common template space for statistical analyses. Lesions traced before 2006 used the MAP-3 method. The MAP-3 method involves the manual tracing of lesion borders on a template brain using the lesion depicted in an MRI scan as a guide and has been previously described.^24,25^ With improvements in automated methods for transforming brains to a common space, lesions traced after 2006 were manually traced on native T1-weighted scans with FMRIB Software Library (FSL) and then transformed to the 1 mm Montreal Neurological Institute (MNI) 152 atlas using non-linear registration and lesion masking techniques available in Advanced Normalization Tools (ANTs).^26^ Because lesions negatively affect the accuracy of the transformation to MNI space, transformations for unilateral lesions were performed using enantiomorphic normalization.^27^ The approach replaces the lesion volume with the voxel intensities from its non-damaged homologue to more closely align the individual’s brain with the template brain. Bilateral lesions were transformed by applying a cost function mask to the lesion volume, which reduces the influence of voxels within the lesion volume on the transformation process.^28^ The spatial transforms were applied to the brain and lesion mask. The anatomical accuracy of the lesion tracing was reviewed in native and MNI space and edited as needed by a neurologist (A.D.B.) blinded to appetite and weight measures.

### Multivariate lesion−symptom mapping

Lesion−symptom mapping analyses were conducted on the BDI-II appetite ratings (Analysis 1—appetite change analysis) and the weight change records (Analysis 2—weight change analysis). Analysis 1 employed partial least squares regression (PLSR) methods and analysis 2, due to the smaller sample size, relied on a proportional subtraction technique.

#### Statistical Analysis 1: appetite change analysis

Appetite assessments were drawn from the BDI-II appetite item, which is a self-report survey designed to evaluate attitudes and manifestations of depression.^29^ The BDI-II captures various dimensions of depression, including affective, cognitive, somatic and vegetative indicators, comprising 21 items. In this study, the scores from patients’ selections within the BDI-II Item 18, Changes in Appetite, were collected and coded as the behavioural data for appetite changes (Table 1). The total score of BDI-II was collected and represented the degree of depression symptoms. If a subject completed more than one BDI-II assessment in the chronic phase post-lesion, the maximum absolute value score among all tests was used for the analysis to capture the time point with the greatest change in appetite.

**Table 1:** BDI-II item 18. Changes in Appetite

0	I have not experienced any change in my appetite.
1a	My appetite is somewhat less than usual.
1b	My appetite is somewhat greater than usual.
2a	My appetite is much less than before.
2b	My appetite is much greater than usual.
3a	I have no appetite at all.
3b	I crave food all the time.

The scale ranges from 0 to 3, with higher scores reflecting greater deviations in appetite, either in the form of appetite reduction (e.g. ‘I have no appetite at all’) or appetite increase (e.g. ‘I crave food all the time’). The appetite decreases (‘a’ items) were changed to negative values for the analysis to create a 7-point scale from +3 to −3.

PLSR was used to model the data using the Iowa Brain−Behaviour Modeling Toolkit in MATLAB.^30^ A direct total lesion volume control (DTLVC) approach was utilized to mitigate effects of lesion size by dividing the lesioned voxels in each patient’s lesion mask by the square root of the lesion volume.^31^ Only brain voxels lesioned in at least 10 patients were included in the analysis to minimize risk of underpowered brain regions contributing to the model. To determine the significance of the inferential model compared to an empirical null distribution of model fits, permutation testing was performed using 1000 permutation iterations. This holistic approach evaluates the statistical significance of the entire map in a single test. Bootstrapping (1000 iterations) was used to estimate 95% confidence intervals on the in-sample model fit and to estimate voxel-wise *z*-statistics that were then used to evaluate the statistical significance of individual voxel-level predictor effects in the multivariate model. A family-wise error corrected threshold of 0.05 was used to correct for multiple comparisons in voxel-level tests. By applying multivariate regression techniques, such as PLSR, this method effectively accounts for complex, distributed lesion−behaviour relationships.

Potential confounds in this lesion−appetite relationship, including severity of motor impairment, time from lesion to appetite assessment and depression severity were evaluated using linear regression. Motor impairment: appetite disturbances have been linked to reduced physical performance and mobility, with one study reporting significant associations between decreased appetite and lower muscle strength, mobility and physical function in hospitalized older adults.^32^ To explore the influence of motor impairment on appetite change, the relationship between the left-hand and right-hand Groove Pegboard Test scores and appetite change score was assessed for a subset of patients with both measures obtained (*n* = 298 and *n* = 300, respectively). Time from lesion to appetite assessment: linear regression was utilized to model the relationship between the number of days from lesion onset to BDI-II test date and appetite change score to evaluate the relationship between reported appetite change and the time elapsed between the lesion and the appetite assessment. Depression severity: to evaluate the influence of depression symptoms on appetite change, linear regression was used to model the relationship between patients’ BDI-II total scores (minus appetite) and the BDI-II appetite change scores.

#### Statistical Analysis 2: weight change analysis

All participant weight records were reviewed, including a review of paper medical records for dates before approximately 2010 and the Epic electronic medical record system after 2010. Weight change was calculated as the per cent change in body weight from W1 to W2, consistent with prior literature using per cent change to minimize confounding by individual differences in pre-lesion baseline weight.^33^

Including all patients with weight change data following the inclusion/exclusion criteria for analysis 2, *n* = 48 patients were available for analysis. Due to the smaller sample size and limited lesion coverage across the brain compared to the appetite change cohort, a PLSR approach was not feasible. Thus, voxel-level mass-univariate proportional subtraction analyses were performed. Patients were binarized into a −1 (weight loss) group and a + 1 (weight gain) group post-lesion. Proportional overlaps were generated for each group by summing binary predictor features across all observations within each group and dividing by the total number of observations in each group. The resulting proportional overlap map for the group coded −1 (weight loss) was then subtracted from the proportional overlap for the group coded +1 (weight gain), resulting in a proportional difference map. To determine the significance of the inferential model compared to an empirical null distribution of model fits, permutation testing was performed using 1000 permutation iterations. The continuous family-wise error (FWE) method was used to determine a single FWE threshold across all voxels at a voxel extent threshold of 1000,^34^ liberally chosen due to the small sample size. However, only 95 voxels survived, failing to surpass the voxel extent threshold. For the surviving voxels, voxel-level permutation tests were performed to estimate uncorrected voxel-level *P*-values, and an exploratory analysis was performed using an uncorrected alpha of 0.05 to identify brain regions showing a trend toward a significant brain−behaviour relationship at uncorrected thresholds.

Potential confounding variables in the lesion−weight loss relationship were again explored with linear regression models, evaluating the relationship between weight loss and motor impairment, depression symptom burden, time elapsed from W1 to W2 and lesion volume. The relationship between appetite change score and weight change score was also explored for the subset of patients with both measures (*n* = 47). Motor impairment was again quantified using performance on the Grooved Pegboard Test for the subset of patients with both this measure and repeatedly prepost recorded weights (*n* = 41). Depression symptoms were quantified using the BDI-II scores after subtracting out the appetite measure and compared to the absolute value of weight change, considering depression is variably associated with both weight loss and weight increase.

A secondary analysis was performed on patients with greater than 5% change in weight^35,36^ following their lesion, in order to highlight brain regions associated with clinically significant levels of weight change (*n* = 23). For this cohort, a proportional subtraction analysis was performed for visualization and comparison with findings from the larger weight change cohort.

## Results

### Patient demographics

**Figure 1:**
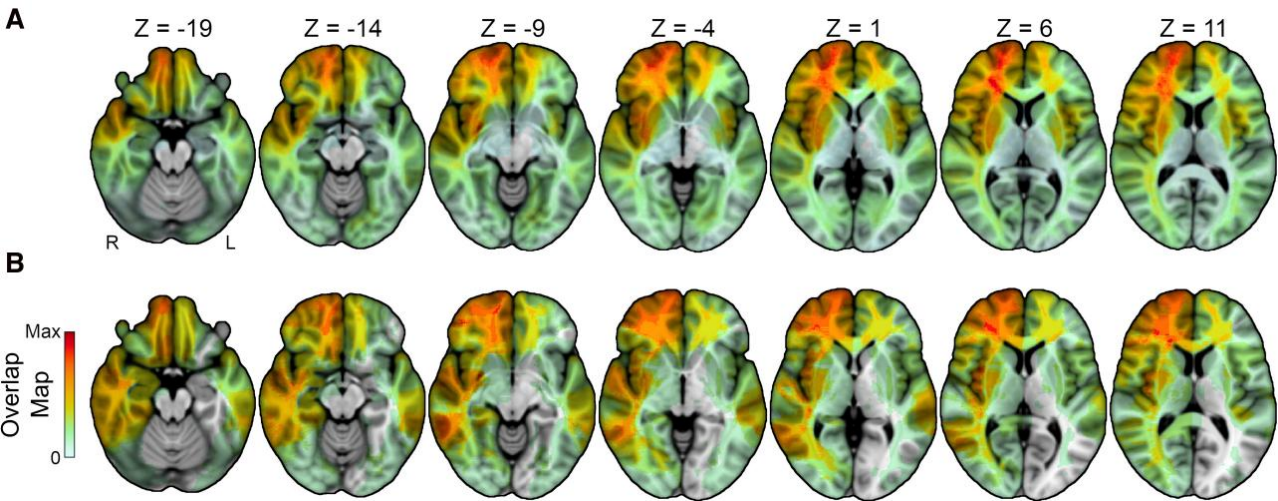
**Overlap maps for the appetite cohort and weight cohort.** (**A**) The axial montage for the appetite change cohort (*N* = 358, Max Overlap = 41). (**B**) The axial montage for weight change cohort (*n* = 48, Max Overlap = 8).

For the appetite analysis (*n* = 358), the average age of patients at the time of the appetite assessment was 48.1 years (*SD* = 17.5). The 178 were male (49.7%) and 352 were White (98.3%). For the weight analysis (*n* = 48), the average age of patients at the time of the appetite assessment was 51.9 years (*SD* = 14.4). The 23 were male (47.9%) and 47 were White (97.9%). Regarding outcomes of interest, 32% of patients reported decreased appetite, 24% reported increased appetite and 44% reported no change after their brain lesion. Similarly, 52% of the weight analysis cohort showed weight loss and 48% showed weight gain in the post-lesion period, with 31% demonstrating > 3 kg weight loss. The average weight change in the entire cohort was −0.8 kg (−0.24% of baseline weight, standard deviation of +/−8.3 kg), consistent with larger population-based data in the post-stroke period.^37^ Additional demographic information can be found in Table 2. Lesion overlap maps were generated for patients in the appetite change and weight change samples, as detailed in Fig. 1.

**Table 2:** Demographics for the appetite and weight cohort

	Cohorts	
Demographics	Appetite change cohort (*n* = 358)	Weight change cohort (*n* = 48)
Age at lesion onset, years (SD)	48.1 (17.5)	51.9 (14.1)
Gender	178 Male (49.7%)	23 Male (47.9%)
Time, lesion to scan, years (SD)	3.6 (7.3)	1.8 (2.0)
Time, lesion to appetite rating, years (SD)^a^	5.1 (8.5)	1.4 (1.8)
Handedness	312 Right (87.2%)	40 Right (83.3%)
Race	352 White (98.3%)	47 White (97.9%)
Education, years (SD)^a^	14.0 (2.4)	14.5 (2.5)
Lesion aetiology	Mixed (62% stroke, 25% resection, 5% SAH^b^, 5% head trauma, 2% encephalitis, 1% other)	Mixed (71% stroke, 23% resection, 4% encephalitis, 2% head trauma)
Directionality of change	32% decreased, 24% increased, 44% no change	52% decreased, 48% increased

^a^Due to limited data availability, age at BDI assessment, exact time from lesion to BDI assessment and education level were not available for one patient in weight change cohort, so only 47 participants’ data were calculated for the time from lesion to appetite rating and the year of education for the weight change cohort.

^b^SAH, subarachnoid haemorrhage.

### Appetite analysis results

**Figure 2:**
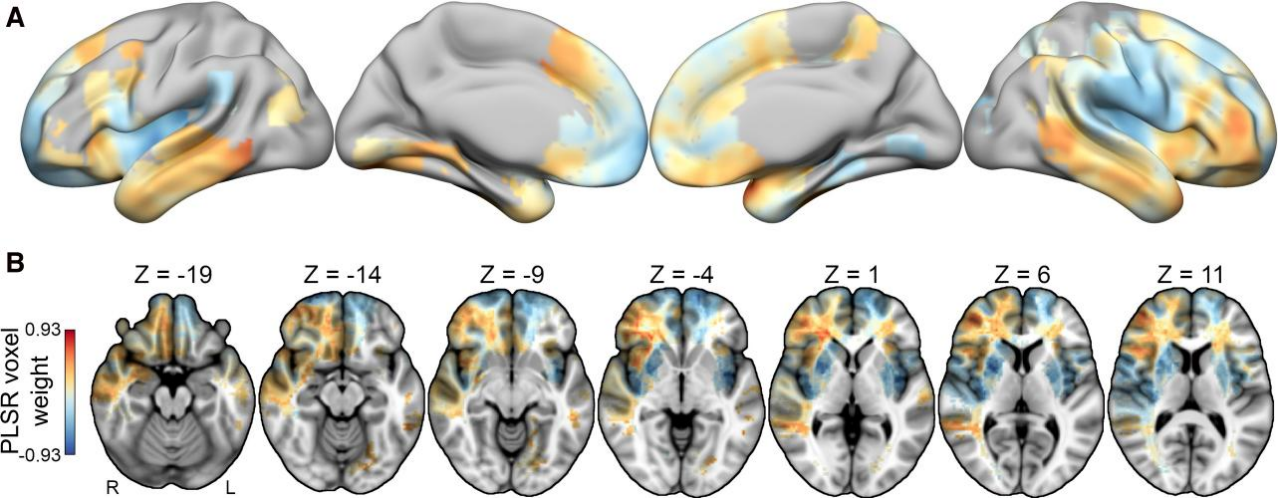
**Lesion sites associated with appetite change.** (**A**) The partial least squares regression analysis revealed a significant multivariate association between lesion location and changes in appetite, as measured by the BDI-II (*N* = 358, permutation *P* = 0.006). The full unthresholded voxel weight map, which encodes the PLSR model coefficients, is visualized on an inflated brain. The surface view shows blue areas representing regions where damage is linked to a decrease in appetite, while orange areas indicate regions where damage is associated with an increase in appetite. (**B**) The axial images further demonstrate the spatial distribution. Regions linked to appetite decrease include the bilateral mid-to-posterior insula, the bilateral putamen and the left frontal pole, while regions associated with appetite increase include the right ventrolateral prefrontal cortex, the right ventromedial prefrontal cortex (vmPFC) and the bilateral temporal lobes.

A multivariate PLSR analysis was used to evaluate the relationship between lesion location and appetite change, with appetite measured with the Changes in Appetite item on the Beck Depression Inventory-II. An inferential model fit to the appetite cohort explained 12.54% of variance in the outcome across the full sample (95% CI: [0.029, 0.222], permutation *P* = 0.006), indicating a statistically significant association between the multivariate pattern of voxel weights encoded in the PLSR model and appetite change scores. However, bootstrap tests evaluating the statistical significance of individual voxel coefficients did not identify any significant voxels at corrected statistical thresholds (all FWE*p* > 0.05). Nonetheless, the voxel weight magnitudes, which reflect the relative contributions of each voxel to the fitted model, indicated that decreased appetite was most strongly associated with damage to the posterior insula, detailed in Fig. 2, and also revealed associations with damage to the bilateral mid-to-posterior insula, the bilateral putamen and the left frontal pole. In contrast, increased appetite was most strongly associated with damage to the right ventrolateral prefrontal cortex, the ventromedial prefrontal cortex and the bilateral temporal cortices. While these regional associations should be understood as descriptions of the relative regional contributions to the full multivariate pattern since individual voxel-level effects did not survive multiple comparisons correction, they nonetheless highlight strong contributions from insular and prefrontal regions to the appetite change model.

Analyses of potential confounding variables showed no significant relationship between motor function and appetite change (linear regression results for left- and right-hand motor function and change of appetite: left hand *n* = 298, *r* = 0.018, *P* = 0.754; right hand *n* = 300, *r* = 0.040, *P* = 0.492). These results suggest that physical motor dysfunction and reduced mobility, as measured with available data for hand function, is not reliably associated with appetite change in this patient sample. Similarly, linear regressions showed no significant associations between appetite change and time elapsed between lesion onset and the appetite assessment (*n* = 351, *r* = 0.08, *P* = 0.134). Furthermore, there is no significant association between appetite change and other depressive symptoms as measured with the BDI-II after removing the appetite score (*n* = 358, *r* = 0.07, *P* = 0.18). To further ensure no effect of depression symptoms on appetite, a secondary analysis was performed, using the same PLSR settings with the addition of confound regression to remove the variance associated with the BDI-II total score (minus the appetite variable) from the outcome variable (appetite change item). The maps were almost identical, with a spatial correlation coefficient of 0.994. One possibility explaining why appetite did not correlate more strongly with BDI-II depression scores is that the total BDI-II scores among the appetite cohort ranged from 0 to 48 (mean = 12.26, SD = 8.77), indicating that most participants fell within the mild-to-moderate range of depressive symptoms, and may thus have had more modest correlations than expected in severe depression or idiopathic major depressive disorder.

### Weight analysis results

**Figure 3:**
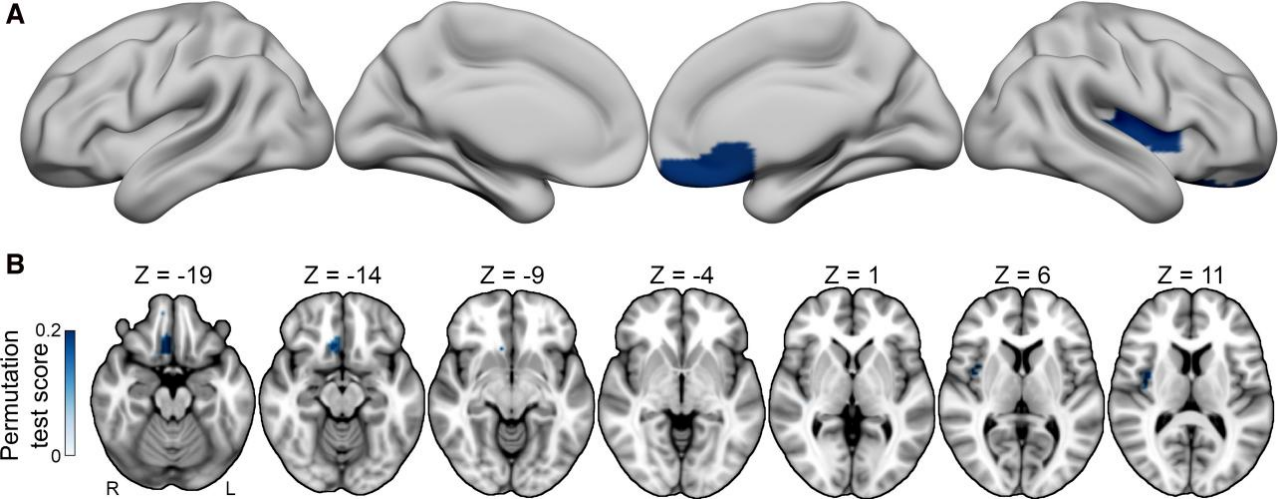
**Regions associated with weight change.** (**A**) A proportional subtraction analysis result demonstrates lesion sites associated with post-lesion weight loss (*N* = 48, uncorrected *P* = 0.02). (**B**) The axial montage further details the spatial distribution of these associations, highlighting the regions associated with weight loss. No voxels associated with weight gain survived permutation testing.

For the analysis of weight change (*n* = 48), a proportional subtraction analysis demonstrated two primary regions that were associated with weight loss post-lesion—the right mid-to-posterior insula and the right ventromedial prefrontal cortex (uncorrected *P* = 0.02). These results as identified with permutation testing are visualized in Fig. 3. These regional findings did not withstand multiple comparisons correction. A secondary analysis on the subset of patients with clinically significant weight change, as defined by prior research,^38^ also employed proportional subtraction, but limited the analysis to patients with greater than 5% change from baseline body weight post-lesion (*n* = 23). Peak findings were identified in similar brain regions—weight loss was associated with lesions to the right mid-to-posterior insula, the left anterior-to-mid insula and the right ventromedial prefrontal cortex. Weight gain post-lesion primarily localized to the right inferior frontal gyrus and the bilateral occipitotemporal regions (Supplementary Fig. 1).

In the weight change cohort, an analysis of potential confounds showed no significant relationship between motor function and weight change (linear regression of left-hand motor function: *n* = 42, *r* = 0.124, *P* = 0.434; right-hand motor function: *n* = 42, *r* = 0.191, *P* = 0.226). Similarly, additional regression analyses showed no significant associations between weight change and non-appetite depression symptoms (*n* = 47, *r* = 0.08, *P* = 0.58), time between weight measurements (*n* = 48, *r* = 0.09, *P* = 0.53) or lesion volume (*n* = 48, *r* = 0.10, *P* = 0.49).

Notably, there was a modest correlation between weight change and appetite change in patients with both measurements, trending towards statistical significance despite the small sample size (*n* = 47, *r* = 0.27, *P* = 0.065) (Supplementary Fig. 2).

### Illustrative case reports of appetite and weight loss following insula lesions

To illustrate the effects of insula lesions on appetite and weight, we present two individual case reports. Both patients provided informed consent for the publication of these case reports and were interviewed approximately 6 months after their strokes.

**Figure 4:**
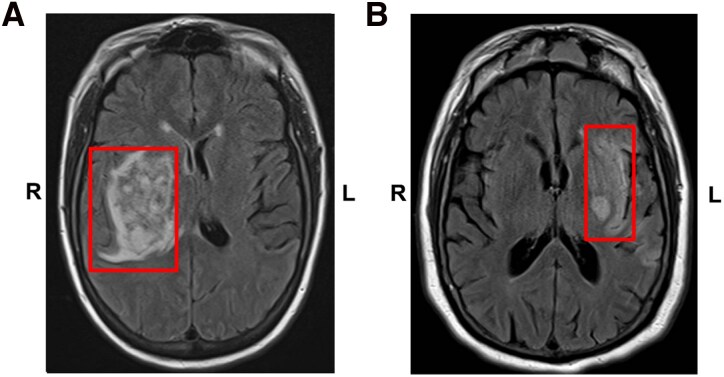
**MRI scans for case examples.** These T2-weighted fluid-attenuated inversion recovery (T2 FLAIR) axial slice MRI scans demonstrate the lesions from our two patient case examples, affecting the insula and basal ganglia on the right (A, Case 1) and the left (B, Case 2) hemisphere, respectively. Lesion areas are outlined in red, and ‘R’ and ‘L’ indicate the right and left hemispheres, respectively.

Patient 1 was a right-handed white female who suffered a right insula and basal ganglia lesion due to a haemorrhagic stroke in her early thirties (Fig. 4, left). After the stroke, she experienced left-sided weakness, sensory changes and dysarthric speech as expected for a lesion in this region, which gradually improved over the next 6 months, although with some residual left-sided weakness primarily in the left arm and left leg. Notably, she also developed prominent changes in appetite and associated weight loss.

When interviewed 6 months after her stroke, she described both loss of appetite and change in the taste of food. She recalls these symptoms starting almost immediately after the stroke, and certainly within the first month, and persisting. She reported that ‘food is not appealing’. Food overall had less taste or no taste, and she said that all foods ‘taste like cardboard; my taste buds have changed for all foods’. She used to eat fast food for every meal, three times per day, and found it very rewarding and enjoyable. Now she may eat fast food once per week and has to force herself to eat. Other pleasurable foods, such as bite-size pizzas from Walmart, were no longer enjoyable. Over the 6 months post-lesion, she had an unintentional 20.4 kg weight loss (24% decrease from baseline) documented.

The history is complicated by the patient having significant immobility for a portion of her recovery, as well as associated mood changes post-stroke including irritability, hopelessness, anxiety, fatigue, crying spells, loss of libido and loss of interest in other pleasurable activities. However, the taste changes and specific loss of interest in food were prominent and occurred on a more rapid time-scale post-stroke; the patient attributed many of her mood symptoms to the stress of managing her limitations post-stroke, but not her appetite change.

Patient 2 was a right-handed white male who suffered a left insula, caudate and putamen lesion due to ischaemic stroke of the left middle cerebral artery in his early seventies (Fig. 4, right). After the stroke, he experienced speech difficulties and right-sided motor weakness as expected for a lesion in this region, which gradually improved over the next 6 months. Notably, he also developed prominent appetite changes by his and his wife’s report.

When interviewed 6 months after his stroke, his appetite remained generally decreased. He reported a new intolerance to certain food textures. For example, the patient reported that prior to his stroke, Cheetos were his favourite food. After the stroke, he completely stopped eating them, stating they are ‘too crunchy’, and the texture is aversive to him. Due to similar changes in the experience of the taste and texture of foods, he reported that he had lost interest in eating cold cereal or drinking coffee, two other daily food preferences prior to the stroke. In addition to the change in his eating experience, he also reported a general diminishment of his appetite. He stated he would not get hungry but would often eat out of habit. He and his wife reported that he would eat smaller portions and a wider variety of foods than he used to eat. This translated into a loss of weight. At the time of the interview, he had lost 3 kg (3.7% decrease from baseline) unintentionally.

This history is certainly complicated by several factors including concomitant motor and speech changes, damage to other relevant brain regions beyond the insula (namely, the caudate) and report of some depressive symptoms. Regarding his mobility, the patient endorsed gradually improving right upper and lower extremity weakness, yet with some continued functional limitations. He also reported some depressive symptoms, often related to functional impairments (e.g. he lost interest in playing computer games due to difficulty with the computer mouse and fatiguing more quickly). However, he stated he could still enjoy activities when he did them and he did not endorse sufficient symptoms to warrant a clinical diagnosis of a mood disorder. He and his wife both felt his appetite changes were borne more out of a change in his food preferences and experience of eating than due to changes in his mood or physical limitations.

This level of subjective detail is not available for the majority of patients in this study; however, these cases provide a potential framework and set of hypotheses for how insula damage may contribute to appetite and weight change, which could be subsequently followed up in future work. Specifically, these case reports suggest insula damage may affect eating behaviour by altering both one’s gustatory processing and also reward processing typically associated with the pleasurable experience of eating.

## Discussion

We identified a significant association between multivariate lesion patterns and appetite change, and characterized regional associations between damage and weight change. The right mid-to-posterior insula showed the strongest regional associations with decreased appetite and weight loss, representing a convergent finding across analyses. In contrast, other insular regions showed divergent effects: for example, the left anterior-to-mid insula contributed primarily to weight loss but not appetite change, while left mid-to-posterior insula and the left frontal pole contributed to decreased appetite but were less associated with weight loss. The prefrontal cortex and striatum also featured prominently in model, with some qualitative convergent findings in the right ventromedial prefrontal cortex and bilateral striatum which did not reach statistical significance for the weight change sample.

### The insula and its involvement in appetite and weight regulation

As a highly integrated area in the brain, the insula and its subregions play a vital role in multimodal information processing relevant to appetite, which thus offers a potential explanation for one of the key findings in this study. In particular, subregions of the insula are involved in the processing and integration of taste,^39,40^ smell,^41^ texture,^40,42^ fat content^42^ and interoception.^43^ The peak finding in our analyses involves both the granular and dysgranular regions of the mid-to-posterior insula (peak findings in subregions dysgranular insular area 4 [Id4]—primary convergent finding, granular insular area 2 [Ig2]—appetite decrease peak and dysgranular insular area 6 [Id6]—weight loss peak—in the Julich Brain Atlas^44^ [https://atlases.ebrains.eu]), regions known for somatosensory and chemosensory processing, such as for gustation and olfaction.

Previous research shows that the mid-to-posterior insula plays an important role in the perception of food, especially in taste processing and appetite regulation. Direct electrical stimulation of this region in epilepsy patients with implanted intracranial electrodes can induce gustatory and olfactory percepts,^45^ and insular damage has been associated with both appetite and taste dysfunction.^46,47^

The mid-to-posterior insula not only processes taste information, but also integrates food-related visual and olfactory stimuli,^48^ and may integrate these perceptions with higher-order reward processing and salience regions in the anterior insula.^49^ These regions show significant and distinct activation to different taste stimuli such as sweetness or bitterness,^41^ food texture (e.g. hardness or viscosity)^41^ and food fat content (high-fat > low-fat),^41^ as well as to food-related visual information (such as the appearance of food) and olfactory information (such as the aroma of food). Altering these processes via insular damage could therefore affect an individual’s desire for food and ability to regulate appetite.^48^

Finally, insular dysfunction could lead to changes in appetite and weight via disruption of interoceptive processes. Interoception refers to the brain’s perception and integration of incoming signals related to body homeostasis.^43^ The posterior insula plays a central role in this process, integrating sensory information from the body and regulating appetite.^43,50^ Interoceptive processing abnormalities in the dorsal mid-insula in relation to eating and digestion have been identified in patients with eating disorders,^51^ and functional connectivity abnormalities between the mid-insula and the ventral striatum have been identified in obese individuals experiencing hunger relative to healthy-weight individuals.^52^

Thus, the insula, and especially the mid-to-posterior insula, is intimately involved in gustatory, olfactory and interoceptive processes that could significantly impact appetite and eating behaviours when damaged or dysfunctional.

### Mechanistic contributions of the striatum and ventral prefrontal cortex to appetite and weight regulation

Lesions of the putamen, a key component of the striatum, were also associated with decreased appetite and weight loss. The putamen plays a critical role in reward processing and habit formation, including in relation to eating behaviours. Neuroimaging studies have implicated the putamen and dorsal striatum in dopaminergic reward learning associated with eating, for example, correlating feeding-induced dopamine release with meal pleasantness ratings.^53^ Additionally, dopamine production in the striatum has been shown to restore feeding behaviour in dopamine-deficient mice,^54^ emphasizing the critical role of this region in regulating feeding motivation. Clinically, individuals with bulimia nervosa demonstrate reduced activation in the putamen, in addition to the insula and ventral prefrontal regions, in response to taste stimuli, suggesting altered dopamine-related reward processing that could contribute to appetite or weight changes.^55^ Individuals with recurrent binge eating also show alterations in the striatum including abnormal striatal connectivity, reduced dopamine D2/3 receptor binding and microstructural changes.^56^

Finally, increases in appetite in this study were associated with lesions of the right ventrolateral prefrontal cortex (vlPFC) and some portions of the ventromedial prefrontal cortex (vmPFC). The vmPFC and vlPFC are significant in decision-making, reward processing and inhibitory control, all of which are critical for regulating food intake. The ability to resist food cravings or the urge to eat requires proper functioning of neuronal circuits involved in top-down control of conditioned responses predicting food reward. Impairments in prefrontal pathways regulating reward sensitivity, conditioning and cognitive control may therefore contribute to overeating behaviours.^57^ Also, brain-imaging studies comparing obese to lean individuals have reported lower grey matter density in the putamen and frontal regions, including the vlPFC,^58^ suggesting structural differences that may influence eating behaviour and weight regulation. Notably, one lesion study showed patients with vmPFC damage continued to choose foods they no longer found pleasant or rewarding, even though they reported reduced hunger and enjoyment.^59^ This finding suggests a disconnect between internal states and goal-directed reward-seeking behaviour, which may provide an explanation for some of our findings.

### Therapeutic treatments for disordered eating behaviours implicate the insula and prefrontal cortex

These results raise the future possibility of designing therapies for disorders associated with under- or over-eating that target these identified regions of interest. Already, the insula and prefrontal cortex (PFC) have been targeted in the treatment of other problematic habitual behaviours associated with dysfunctional reward seeking. Transcranial magnetic stimulation (TMS) targeting the insula and dorsal prefrontal cortex has resulted in reduced cigarette consumption for nicotine and tobacco users.^60^ TMS protocols targeting the dorsal and ventral PFC have also shown promise for mitigating cravings and substance use in substance use disorders, although it is unclear whether this would translate to reducing food cravings or overeating.^61^ Finally, studies of new GLP-1 analogue weight loss medications have demonstrated reduced activation in the insula and putamen^62,63^ in anticipation of and response to desirable food images. Insula activity changes have been associated with reduced food cravings and regulation of overeating,^64^ suggesting this region may already be a target of currently available therapies and is worthy of further investigation.

### Limitations and future directions

Our study has several limitations. The analysis of appetite change relied on self-report response from the BDI-II, which is subjective and susceptible to recall bias, response bias and individual differences in interpretation. The BDI-II time scale for symptom report inquiries about symptoms over the past 2 weeks, and thus use of this scale could result in capture of post-lesion changes over a narrow time window that could miss longer-term longitudinal fluctuations. If objective food intake measures and more detailed longitudinal appetite and weight information were available, the study findings may be further strengthened.

The absence of a significant association between overall depression severity and appetite change was somewhat unexpected given the known association between depressive symptoms and appetite alterations.^6^ One possible explanation is that appetite disturbance may only emerge in more severe depression and may be more common in idiopathic major depressive disorder, whereas our sample may have had elevated BDI-II scores due to other post-stroke symptoms that were not representative of a major depressive episode. Additionally, BDI scores in our sample were predominantly in the mild-to-moderate range, which may have restricted the variability needed to detect such an effect. Furthermore, because appetite change can manifest in either direction in depressive disorders, the heterogeneous nature of this symptom may have obscured a clear relationship.

Although we evaluated the relationship between motor function and appetite/weight change using the Grooved Pegboard Test and showed no significance, it is possible that the Grooved Pegboard Test, as a test of hand manipulative dexterity, may be insufficient for ruling out a relationship between whole-body mobility and appetite/weight change. Broader mobility measures and other metrics of functional impairment should be considered for better evaluating this relationship in future datasets. For the appetite change analysis, the effect size estimates could be optimistically biased as the PLSR model was fit to the full dataset, and so validating these findings in independent datasets will be crucial. For the weight change analysis, the sample size was relatively small, which limited our lesion coverage and thus the available analyses and statistical power. This limitation necessitated the use of proportion subtraction with a mass univariate approach rather than more advanced multivariate techniques. Lesion coverage was also notably limited or entirely absent in the hypothalamus and brainstem, regions with evidence for mediating feeding behaviour which could not be investigated with adequate power in this study. Finally, our sample was predominantly White, and thus generalizability to other demographic groups is unclear. The association between appetite and weight change is promising nonetheless, and the primary regions of interest from the appetite analysis were recapitulated in the weight change analysis.

### Conclusion

This large-scale lesion symptom mapping study of appetite identified brain regions associated with appetite changes (increase or decrease) following a lesion. The peak area associated with appetite decrease in the lesion-symptom mapping model was the insula, most prominently the right posterior insula. Appetite increase was associated with a model that included lesions to the ventrolateral and ventromedial prefrontal cortices. In a smaller cohort of patients with weight records in close temporal proximity to and following the lesion, convergent findings identified the right posterior insula as a region associated with weight loss post-lesion; convergent but non-significant findings were also seen in the prefrontal cortex and putamen. Disparate trends were seen in the ventromedial prefrontal regions (increased appetite yet weight loss post-lesion). A wide literature supports the involvement of these regions in eating behaviour and taste processing, and case examples from two patients with taste and eating dysfunction following insula damage are presented. Future clinical research should consider these regions when developing biomarkers and therapies associated with abnormal appetite or feeding behaviour.

## Supplementary Material

fcag044_Supplementary_Data

## Data Availability

The data for this study is not publicly available due to privacy and ethical constraints. The corresponding author can provide the data for this study upon reasonable request.
