# Supplementary material for: Lesions involving the insula are associated with reduced appetite and weight loss
Source: Brain Commun. 2026 Feb 25;8(1):fcag044. doi: 10.1093/braincomms/fcag044 (PMC12933212; doi:10.1093/braincomms/fcag044)
Supplement: fcag044_Supplementary_Data [file fcag044_supplementary_data.docx]

# Supplementary material


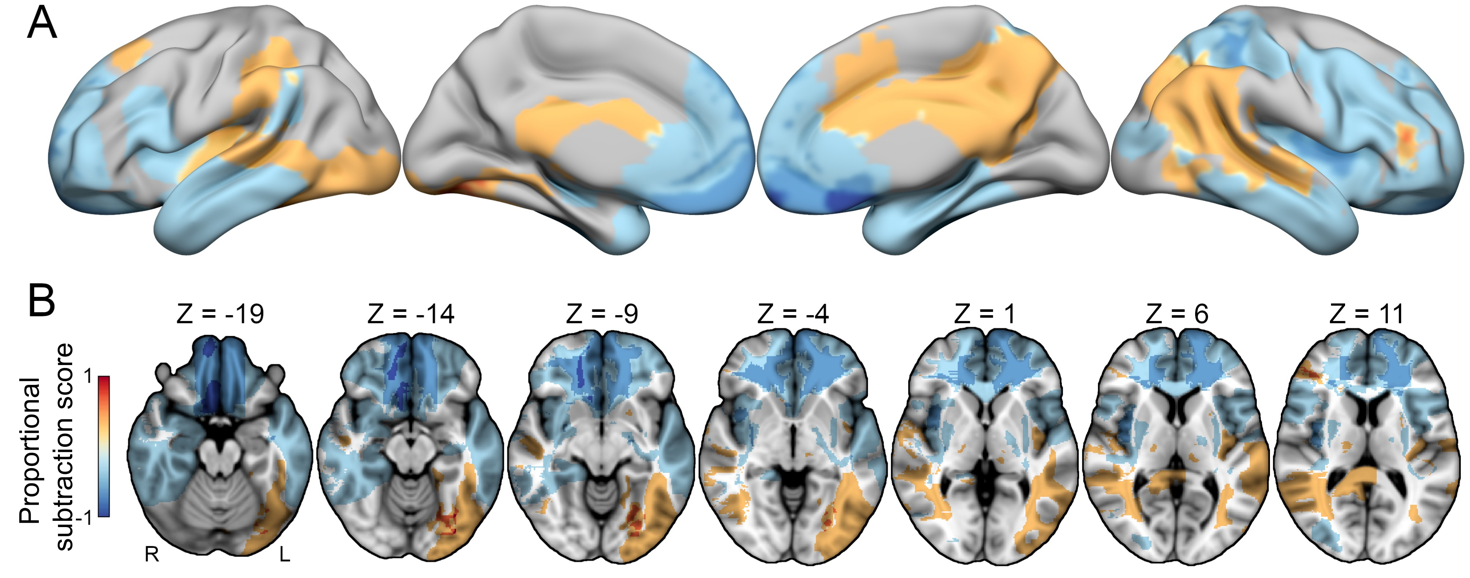


**Supplementary Figure 1 Item 18. Proportional subtraction analysis of weight changes post-lesion.** (**A**) The proportional subtraction analysis, conducted on a sample with more than 5% weight change from baseline (N = 23), revealed key regions associated with weight changes post-lesion. The blue areas represent regions linked to a decrease in weight, while the yellow to orange areas indicate regions associated with an increase in weight. (**B**) The axial montage further details the spatial distribution of these associations. As shown in the figure, weight loss was linked to lesions in the right mid-to-posterior insula, the left anterior-to-mid insula, and the bilateral ventromedial prefrontal cortex, while regions associated with weight gain post-lesion primarily localized to the right inferior frontal gyrus and bilateral occipitotemporal regions.

**
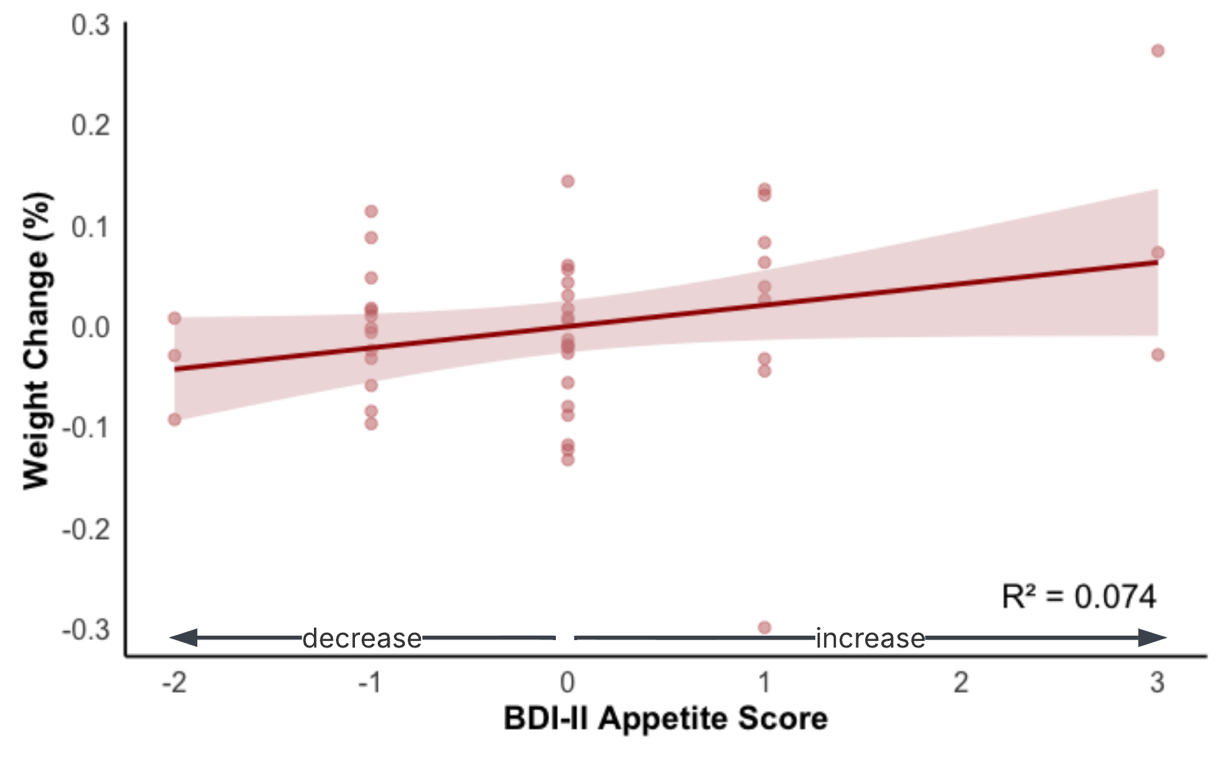
**

**Supplementary Figure 2 Association between weight change and BDI-II appetite score for patients with both measures** (N = 47). The x-axis represents changes in appetite, with values left of zero indicating decreased appetite and values right of zero indicating increased appetite. The y-axis represents post-lesion weight change measured as percentage change from baseline weight. Each data point represents an individual participant’s weight change plotted against their BDI-II appetite change score. The results of a Pearson correlation analysis suggest a tendency for greater weight change (measured as a percentage change from pre-lesion weight) to be associated with higher BDI-II appetite scores (*R^2^* = 0.074, *r* = 0.27, *P* = 0.065).
